# Projections of leaf turgor loss point shifts under future climate change scenarios

**DOI:** 10.1111/gcb.16400

**Published:** 2022-09-05

**Authors:** Enrico Tordoni, Francesco Petruzzellis, Azzurra Di Bonaventura, Nicola Pavanetto, Martina Tomasella, Andrea Nardini, Francesco Boscutti, Fabrizio Martini, Giovanni Bacaro

**Affiliations:** ^1^ Department of Life Sciences University of Trieste Trieste Italy; ^2^ Institute of Ecology and Earth Science University of Tartu Tartu Estonia; ^3^ Department of Agricultural, Food, Environmental and Animal Sciences University of Udine Udine Italy; ^4^ Independent scholar Trieste Italy

**Keywords:** climate change, drought tolerance, ecological plasticity, hydraulic traits, leaf turgor loss point, threatened species

## Abstract

Predicting the consequences of climate change is of utmost importance to mitigate impacts on vulnerable ecosystems; plant hydraulic traits are particularly useful proxies for predicting functional disruptions potentially occurring in the near future. This study assessed the current and future regional patterns of leaf water potential at turgor loss point (Ψ_tlp_) by measuring and projecting the Ψ_tlp_ of 166 vascular plant species (159 angiosperms and 7 gymnosperms) across a large climatic range spanning from alpine to Mediterranean areas in NE Italy. For angiosperms, random forest models predicted a consistent shift toward more negative values in low‐elevation areas, whereas for gymnosperms the pattern was more variable, particularly in the alpine sector (i.e., Alps and Prealps). Simulations were also developed to evaluate the number of threatened species under two Ψ_tlp_ plasticity scenarios (low vs. high plasticity), and it was found that in the worst‐case scenario approximately 72% of the angiosperm species and 68% of gymnosperms within a location were at risk to exceed their physiological plasticity. The different responses to climate change by specific clades might produce reassembly in natural communities, undermining the resilience of natural ecosystems to climate change.

## Graphical abstract

Plant hydraulic traits are useful proxies for predicting functional disruptions caused by climate change potentially occurring in natural ecosystems in the near future. We assessed the current and future regional patterns of leaf water potential at turgor loss point (Ψ_tlp_) by measuring and projecting the Ψ_tlp_ of 166 vascular plant species (159 angiosperms and 7 gymnosperms) across different habitats. We predicted a consistent shift of Ψ_tlp_ toward more negative values especially in low‐elevation areas, and our simulations highlighted that the majority of species within a given location were at risk to exceed their physiological plasticity.

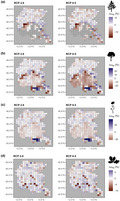

## 1 INTRODUCTION

Anthropogenic climate change is one of the greatest modern global challenges, with drastic consequences for society and nature conservation (Fedele et al., 2019). Among these challenges, the global biodiversity loss is a prominent aftermath of climate‐induced ecosystem disruption (Trisos et al., 2020; Urban, 2015). One of the consequences of climate change is altered precipitation patterns, which result in increased severity, frequency and duration of drought events (IPCC, 2021). These events might promote major ecosystem reorganizations through phenotypic and genotypic acclimation/adaptation, species migration, and local extinctions (Batllori et al., 2020; Fei et al., 2017; Gezon et al., 2016; Martinez‐Vilalta et al., 2019; Trugman et al., 2020). The increase in drought frequency and intensity is associated with declining forest productivity (Poorter et al., 2017) and pulses of tree mortality, mainly caused by hydraulic failure (Hammond et al., 2019; Senf et al., 2020), even in biomes which are less prone to these events (i.e., temperate and tropical humid forests; Phillips et al., 2009; Powers et al., 2020). On this basis, anticipating climate change impacts on plant communities and improving our understanding of their potential vulnerabilities among different clades needs to improve (Choat et al., 2012). This holds true particularly for plant communities growing in terrestrial biomes highly vulnerable to these changes, such as alpine forests and Mediterranean vegetation (Giorgi, 2006; Seddon et al., 2016).

During drought events, soil moisture declines in response to low precipitation coupled with high evaporative demand. One of the first responses of plants to water shortage is stomatal closure, which limits water loss, maintains adequate cell hydration, and prevents water potential (Ψ) from dropping to values that induce cell turgor loss or xylem embolism, which may ultimately lead to plant damage and death (Choat et al., 2018; Zhu et al., 2018). Leaf water potential at turgor loss point (hereafter turgor loss point, Ψ_tlp_) is a measure of the capability of plants to maintain cell turgor under water stress, and is related to stomatal kinetics and water‐use efficiency (Brodribb et al., 2003; Petruzzellis et al., 2021). This is strongly controlled by phylogeny and growth forms because woody angiosperms have better optimized water‐use efficiency with respect to older lineages and herbaceous angiosperms (Brodribb et al., 2003, 2009), even though recent evidence has challenged the view of woody species being more drought resistant than herbaceous ones (Lens et al., 2016). Ψ_tlp_ is a good proxy of species‐specific drought tolerance (Bartlett, Scoffoni, & Sack, 2012; McGregor et al., 2021; Zhu et al., 2018), and lower Ψ_tlp_ is based on osmoregulation, namely increased cell solute content that lowers the osmotic potential at full turgor (π_0_) (Bartlett, Scoffoni, & Sack, 2012). Lower Ψ_tlp_ allows plants to better tolerate water shortage while maintaining positive carbon balance and preventing cellular damage (Blackman et al., 2010; Brodribb et al., 2003), thus ensuring better chances to survive under intense and/or prolonged drought (Nardini et al., 2013). The intimate relation between Ψ_tlp_ and drought tolerance has been reported for several species across different biomes (Bartlett et al., 2014; Zhu et al., 2018) and water availability (Medeiros et al., 2019; Petruzzellis et al., 2021; Tordoni, Petruzzellis, et al., 2020). Turgor loss point is typically lower in arid and saline habitats such as saltmarshes (Rosas et al., 2019; Tordoni et al., 2019), although recent research detected a higher survival in herbaceous species characterized by higher Ψ_tlp_ in temperate grasslands (Sun et al., 2020). Similar to drought tolerance, plants can resist frost damage by lowering π_o_ (Beck et al., 2007; Charrier et al., 2021 and references therein), because ice formation in the apoplast typically leads to cellular dehydration.

In recent years, plant water‐related traits, like Ψ_tlp_ or the vulnerability to xylem embolism (P_50,_ the xylem water potential inducing 50% loss of xylem hydraulic conductivity; Maherali et al., 2004) have been frequently used to predict vegetation responses to climate change (Kunert et al., 2021; Larter et al., 2017; Laughlin et al., 2020; Maréchaux et al., 2018; Oliveira et al., 2019; Trueba et al., 2017). Plant water status influences photosynthesis and ecosystem productivity, which in turn is linked to carbon and hydrological cycles (Adams et al., 2017; Brodribb, 2009; Choat et al., 2018). Thus, the inclusion of such mechanistic traits when modelling vegetation responses to climate is crucial to capture reliable trends and improve projections of climate change impacts (Anderegg et al., 2019). In addition, given their high degree of conservatism within species, these parameters are very useful for trait‐based modelling (Fuchs et al., 2021). Recent methodological advancements (Bartlett, Scoffoni, Ardy, et al., 2012; Griffin‐Nolan et al., 2019; Petruzzellis et al., 2019) allow faster measurements of Ψ_tlp_ compared with traditional methods (e.g., pressure–volume curves), thus enabling its quantification in larger species pools.

To the best of our knowledge, no previous study has provided a spatialization of Ψ_tlp_ at a regional scale. Most importantly, the possible use of Ψ_tlp_ to model vegetation shifts under climate change projections have not been previously tested. This study reports measurements of Ψ_tlp_ for 166 vascular plants (159 angiosperms and 7 gymnosperms). The spatial distribution of this trait was assessed in a grid covering a large climatic gradient spanning from Mediterranean to alpine areas in NE Italy. Additionally, the possible future spatial distribution of Ψ_tlp_ in two climate scenarios was modeled under low and high concentration of greenhouse gases (representative concentration pathway, RCP 2.6 and RCP 8.5, respectively). Specifically, this study aimed to (a) assess the spatial patterns of Ψ_tlp_, (b) identify the main climatic determinants of Ψ_tlp_ variation, and (c) predict future changes in Ψ_tlp_ distribution based on climatic projections up to 2100. It was hypothesized that a different spatial pattern of Ψ_tlp_ would occur for angiosperms and gymnosperms, as well as more negative values of Ψ_tlp_ in areas characterized by high frequency of drought and/or frost events (i.e., mountain valleys, coastal areas).

## 2 MATERIAL AND METHODS

### 2.1 Study area

The study was carried out in Friuli Venezia Giulia region (FVG, geographical limits between 45°35′ N and 46°39′ N, and 12°20′ E and 13°07′ E). The territory is predominantly mountainous (42.5% of the total area) spanning from 0 to 2780 m above sea level (a.s.l.) and is dominated by Carnic and Julian Alps in the northern part. The southern part is characterized by wide plains degrading toward the Grado‐Marano Lagoon and the Adriatic Sea. The South‐eastern part is dominated by classical Karst (Figure S1). Approximately 90% of the region has a temperate climate, the remaining 10% being assigned to continental and polar climates (ARPA FVG, 2018; Cicogna et al., 2018). Mean annual temperatures are higher in the coastal range (14.5–15.5°C), coupled with the lowest mean annual precipitation (ca. 1000 mm; http://www.arpa.fvg.it, accessed July 2021). Mean annual temperature of the alpine sector is approximately 9°C, with minimum temperatures reaching −25°C, while rainfall is abundant and ranges from 1500 to 3000 mm/year in the Julian Alps. Due to such environmental and climatic heterogeneity, FVG hosts a remarkable variety of habitats and is an important hotspot of endemic plant diversity (Tordoni, Casolo, et al., 2020). Vegetation type in the study area ranges from alpine grasslands and tundra formations to coniferous forests on the summits of the alpine sector, replaced by mixed‐forest or beechwoods and broadleaved forests at lower elevations. The lowlands are intensively exploited for crop growth, even though some seminatural patches characterized by grasslands, alkaline fens, and marshes do still occur in the area. The southern coastline and the lagoon are dominated by halophyte and psammophyte communities, while the vegetation of the typical Karst is mainly composed by xero‐thermophilus bushes and small trees, intermixed with large patches of *Pinus nigra* plantations.

### 2.2 Species distribution

Data on spatial distribution of the different species are based on an ongoing survey project aimed at producing the new vascular plant atlas for the FVG Region (personal communication, January 2022). The database includes more than 280,000 records of vascular plants, collected in the period 1980–2020. Main sources of data are represented by field campaigns, herbarium specimens (both historical and recent) and literature records. The region was divided into 270 Operative Geographic Units (hereafter OGUs), each of 3′ of latitude × 5′ of longitude (ca. 5.5 × 6.5 km), following the Central European grid for floristic surveys (Ehrendorfer & Harmann, 1965). However, because some OGUs on the borders have incomplete data, they were excluded from the data set, thus maintaining 264 OGUs for analysis. Species' names were standardized according to the updated checklist of Italian vascular flora (Bartolucci et al., 2018).

### 2.3 Climatic data

A set of climatic predictors was obtained from climate simulations performed by the International Center of Theoretical Physics (ICTP) within the EURO‐CORDEX (https://www.euro‐cordex.net) and MED‐CORDEX (https://www.medcordex.eu) projects, aimed at providing improved regional climate change projections for Europe. Simulations involved combinations of Global Circulation Models (GCMs) and Regional Circulation Models (RCMs). For this study, the following couples of GCM–RCM were selected: HadGEM2‐ES‐RACMO22E, EC‐EARTH_RACMO22E, EC‐EARTH_CCLM4‐8‐17. Data are available at daily temporal resolution, with spatial resolution of 0.10° (ca. 11 km) and can be downloaded from the website of the environmental protection regional agency (https://www.osmer.fvg.it). The data set consists of 16 climatic parameters (including precipitation, surface temperature, and evaporation), encompassing the historical period of 1970–2005, along with simulations for the period 2006–2100 under three RCP scenarios (RCP 2.6, RCP 4.5, and RCP 8.5). For this study, only RCP2.6 and RCP 8.5 were considered. From these climatic data, a set of six biologically relevant variables were derived (sensu https://www.worldclim.org) to quantify both average climate conditions and climatic limits, particularly in relation to drought or frost events. The selected variables were: 95th percentiles of average temperature (BIO1.95, °C), temperature seasonality (BIO4, °C), annual consecutive frost days where temperature was ≤0°C (CFD.ann, n° days), annual consecutive dry days where precipitation was <1 mm (CDD.ann, n° days), 5th percentiles of cumulate annual precipitation (BIO12.5, mm), and precipitation seasonality (BIO15, %). Data were extracted and processed using “ncdf4” (Pierce, 2019) and “raster” (Hijmans, 2021) R packages. For each Operative Geographic Unit, the weighted average value of the climatic variable within each grid cell was extracted based on occupancy within the OGU. Finally, the average value for the historical period was calculated, and for future projections the average value for the period 2080–2100 was computed.

### 2.4 Sampling design and turgor loss point measurement

In total, samples from 166 vascular plant species were collected, representing the most abundant taxa in the regional flora (see Table S1 for the species list). Specifically, 159 angiosperms were sampled which represent approximately 8% of the angiosperms in the region (10% for herbaceous species and 20% for woody species, respectively), and 7 gymnosperms encompassing 39% of all gymnosperm species occurring in the study area. Measurements were performed on three individuals for both herbaceous (one replicate per individual) and woody species (three replicates per individual). Measurements for 124 species were obtained in a previous sampling campaign (see Petruzzellis et al., 2021), while the remaining 73 species were selected following a double‐criterion approach, based on their frequency in the study area and on their representativeness in the regional habitats (ISPRA, 2009), according to the European Habitats Directive (92/43/EEC). In detail, FVG was divided in five areas reflecting the main orographic units (Alps, Prealps, High Plains, Low Plains and coast, classic Karst, Figure S1b). In each area, all species occurring with a frequency ≥90% were selected along with peculiar species characterizing each habitat occurring in each OGU, based on expert assessment (ISPRA, 2009). The sampling campaign was carried out between August and September 2020. Whole individuals of herbaceous plants and 1‐year old twigs of woody plants were sampled. Samples were wrapped in cling film, put in sealed plastic bags with humid paper inside, and maintained in a cool box until processing in the laboratory on the same day of sample collection. Samples were rehydrated overnight with tap water, while covered with a black plastic bag. The following day, one to nine leaves (depending on leaf size) per twig (or per individual in the case of herbaceous species) were sealed in cling film and immersed in liquid nitrogen for 2 min. Leaves remained sealed in cling film to be carefully crushed and stored in sealed plastic bottles at −20°C until measurement. Measurements of π_0_ were done with a dew point hygrometer (WP4, Decagon Devices Inc.) after thawing samples at room temperature for 5 min (10 min for gymnosperms). Finally, π_0_ and Ψ_tlp_ were calculated using equations reported in Petruzzellis et al. (2019).

### 2.5 Spatial patterns of turgor loss point

Considering that a simple average of Ψ_tlp_ values could not provide a reliable estimate of the average species' drought resistance in each grid due to differences in species abundance and representativeness, a four‐step procedure was applied to estimate the weighed value of Ψ_tlp_ in each OGU. The following steps were applied. (1) The relative contribution (%) of each habitat in each OGU was assessed, based on the habitat map (ISPRA, 2009). (2) Each species was associated with each habitat and assigned a weight based on their putative abundance for each habitat according to its ecological optimum (i.e., phytosociological classification, ISPRA, 2009) as follows: dominant species (cover >50%) = 1, co‐dominant (cover <50%) = 0.7, characteristic (common but not abundant) = 0.4, and other characteristic species (uncommon species) = 0.1. (3) The species not present in the grid were filtered out, and the Ψ_tlp_ associated with each habitat (Ψ_tlp_hab_) was computed as a weighted average, using the species' importance as a weight. (4) Finally, following an approach similar to community‐weighted means (Lavorel et al., 2008), Ψ_tlp_hab_ was multiplied by the relative abundance of the habitat to obtain the average value of Ψ_tlp_ over the whole habitat within a grid cell. To effectively remove effects due to phylogeny and growth form, the whole species pool was divided into four subsets (gymnosperms, angiosperms, herbaceous, and woody angiosperms), and for each group Ψ_tlp_ was calculated following the procedure described above.

### 2.6 Statistical analysis

First, one‐way ANOVA was used to test for significant differences in Ψ_tlp_ among different habitats, ensuring that the model's assumptions were met. Post‐hoc multiple comparisons were performed using Tukey's “Honest Significant Difference” method available in “agricolae” (de Mendiburu, 2021) R package. Random forests (RFs) was used to model the spatial patterns of Ψ_tlp_ and the main climatic determinants, as this class of model proved to deal with overfitting and multicollinearity, performing well also in the presence of non‐linear relationships or complex interactions (Cutler et al., 2007; Mansfield et al., 2020). Four RFs were trained for each model using Ψ_tlp_ as a function of climatic variables using “mlr3” (Lang et al., 2019) and “mlr3spatiotempcv” (Schratz & Becker, 2021) R packages. The following parametrization was used to start building the decision trees: sample fraction = 0.6, number of variables to possibly split at each node (*mtry)* = 1, number of trees (*ntree)* = 500, minimal size of the node (*min.node.size)* = 1, which was later tuned using the “paradox” (Lang et al., 2021) R package. Before computing the models, all the climatic predictors were centered and scaled to unit variance. Variable importance was assessed by estimating the average change in root mean square error (RMSE) after variable permutations (*N* = 500) using “DALEX” R package (Biecek, 2018). This method assumes a worsening of the model's performance (i.e., an increase in RMSE) when permuting an important variable caused by a loss in explanatory ability (see Fisher et al., 2019 for more technical details). Partial dependence plots were used to display predictor marginal effects by using “iml” (Molnar et al., 2018) R package.

Several studies showed that ignoring spatial autocorrelation processes can lead to biased conclusions in ecology (e.g., Bacaro et al., 2016; Dormann et al., 2007) or to an excess in model performance (Ploton et al., 2020; Schratz et al., 2019). Accordingly, an internal spatial cross‐validation was performed, creating five partitions (i.e., spatially disjointed subsets), which maximize spatial distance between training and validation with respect to random partitioning (Lovelace et al., 2019). A nested resampling approach was used (Schratz & Becker, 2021), based on two sampling processes (outer vs. inner) where the first one evaluated model performance, and the latter one tuned model hyperparameters in each partition. Due to its computational cost, five folds were selected with 25 repetitions for the outer resampling, while five folds and 50 evaluations of model settings were chosen for inner resampling.

The values of Ψ_tlp_ under future climatic projections were then predicted, and the relative deviation (%) from the measured value in the grid cells were calculated as follows: ΔΨ_tlp_ = [([predicted Ψ_tlp_–measured Ψ_tlp_]/measured Ψ_tlp_) × 100]. Furthermore, spatial uncertainty of model predictions was assessed by estimating the congruence, evaluated on the basis of the coefficient of variation among predicted Ψ_tlp_ across the three different climatic models for each group in each scenario. Maps were realized using “tidyverse” (Wickham et al., 2019), “scico” (Pedersen & Crameri, 2020), “sf” (Pebesma, 2018), and “patchwork” (Pedersen, 2020) R packages. All analyses were performed in R 4.1.0 (R Core Team, 2021).

### 2.7 Threatened species based on plasticity simulations

To evaluate the number of species potentially threatened by climate change, for each grid cell the predicted Ψ_tlp_ was first assessed by averaging the predictions deriving from the RFs. By assuming that the measured Ψ_tlp_ of each species represents the median value of its seasonal plasticity, two simulations were performed (high and low plasticity, respectively) where the species were deemed capable to adjust their Ψ_tlp_ by 0.44 and 0.22 MPa (Bartlett et al., 2014). Finally, the relative number of species in each OGU (expressed as %) whose plasticity range was lower, higher, or does not include the predicted Ψ_tlp_ of the OGU was estimated. This simulation was performed for pooled angiosperms and gymnosperms, considering only the high‐emission scenario (RCP 8.5).

## 3 RESULTS

**FIGURE 1:**
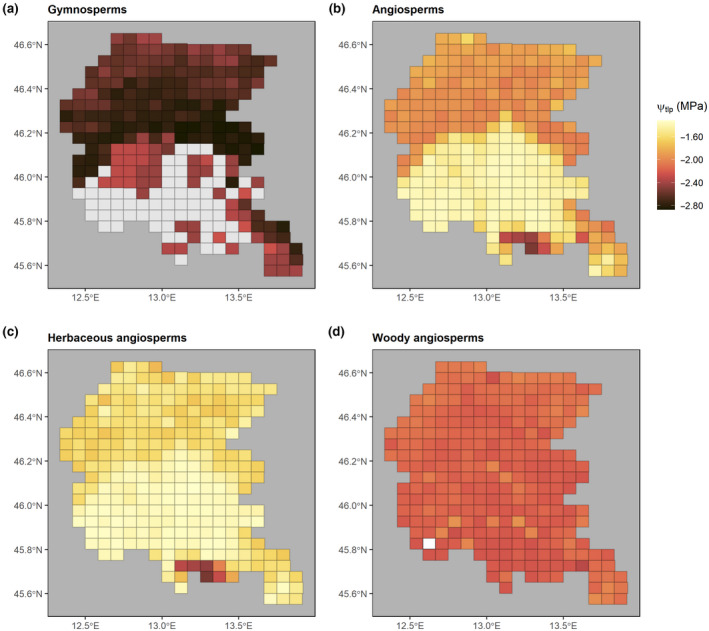
Spatial patterns of Ψ_tlp_ (MPa) in (a) gymnosperms, (b) angiosperms, (c) herbaceous angiosperms, and (d) woody angiosperms. Cell size is 3′ × 5′ (lat/long; ca. 5.5 × 6.5 km). Cells with no data are represented in light gray.

Different habitats were characterized by a significant degree of variation in the observed Ψ_tlp_ (one‐way ANOVA test *F*_[13,262]_ = 7.55, *p* < .001). Notably, saltmarshes and Mediterranean evergreen oak woodlands hosted species with lower Ψ_tlp_ compared with other habitats. Crops, mesophilous grasslands and freshwater habitats generally hosted species less resistant to drought (higher Ψ_tlp_) (Figure S2). A clear and consistent spatial pattern in Ψ_tlp_ was observed between angiosperms and gymnosperms (Figure 1), despite a substantial difference in the magnitude of variation, exceeding 1 MPa in some locations. Gymnosperms (Figure 1a) showed more negative values in the Prealps and in some portions of the Karst area (see Figure S1 for a detailed overview of the main orographic units in the region). Consistently, angiosperms (Figure 1b) displayed more negative values mainly in the Prealps and Alps and in the south‐eastern area (i.e., the lagoon and the Karst, respectively). Within angiosperms, herbaceous species showed a pattern of Ψ_tlp_ variation consistent with that observed for the whole pool of angiosperms, with more negative values in the alpine sector and the Karst. Herbaceous species showed larger variation of Ψ_tlp_ than woody angiosperms, whose drought resistance was evenly distributed in the region (Figure 1d).

### 3.1 Drivers of turgor loss point variation and future shift projections

**FIGURE 2:**
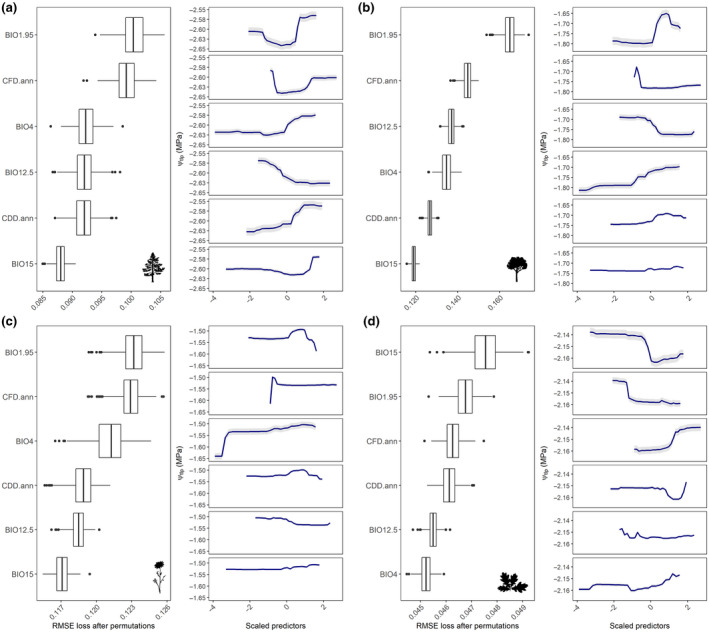
Variable importance (left panel) and marginal effects (right panel) related to the model HadGEM2‐RACMO22E for (a) gymnosperms, (b) angiosperms, (c) herbaceous angiosperms, and (d) woody angiosperms. Boxplots showed variable importance ranked by the RMSE loss after permutations, while solid lines are marginal effects (mean ± 2 SE). BIO1.95, 95th percentiles of average temperature; BIO4, temperature seasonality; CFD.ann, annual consecutive frost days where temperature was ≤0°C; CDD.ann, annual consecutive dry days where precipitation was <1 mm; BIO12.5, 5th percentiles of cumulate annual precipitation; BIO15, precipitation seasonality. Please note that all predictors have been centered and scaled to unit variance. Information related to the other models is available in Figures S3 and S4. Silhouettes were retrieved from http://phylopic.org.

All RFs showed good performance in predicting the Ψ_tlp_ with an average *R* ^2^ ≈ 0.49 and an RMSE ≈ 0.11 across the different models (Table S2). For sake of readability, only one model is reported in the main text, which best depicts the overall trends in the data set (Figure 2), but information related to the other models are available in Figures S3 and S4.

**FIGURE 3:**
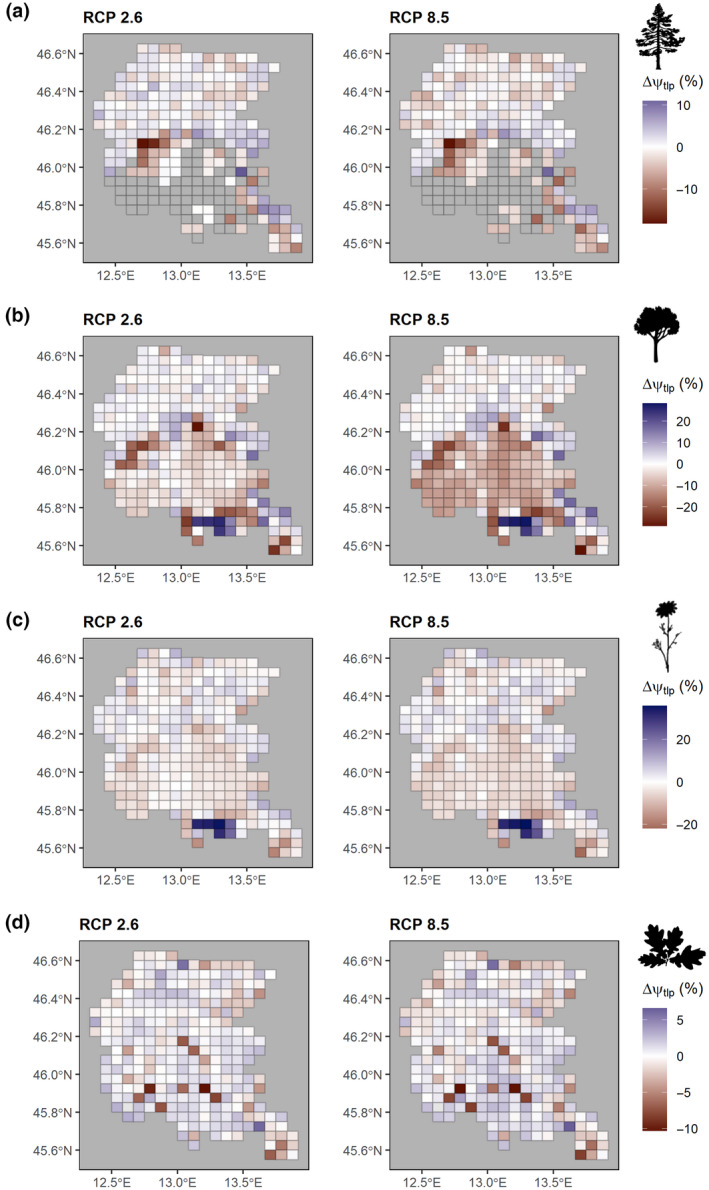
Projections of turgor loss point shifts (ΔΨ_tlp_, %) based on two RCP scenarios (RCP 2.6 and RCP 8.5) computed on model HadGEM2‐RACMO22E for (a) gymnosperms, (b) angiosperms, (c) herbaceous angiosperms, and (d) woody angiosperms. Brown tones indicate a shift toward more negative values of Ψ_tlp_ while bluish tones denote a shift toward higher values of Ψ_tlp_. Silhouettes were retrieved from http://phylopic.org.

For all groups, except for woody angiosperms, the most important variables were temperatures (BIO1.95) and consecutive frost days (CFD.ann), while precipitation seasonality (BIO15) was important only for woody angiosperms (Figure 2d). In the other climatic models, consecutive dry days (CDD.ann) and the 5th percentiles of cumulate annual precipitation (BIO12.5) also emerged as important predictors of the spatial variation of Ψ_tlp_ (Figure S3). Notably, all the models converged in predicting consistent shifts of Ψ_tlp_ with relatively low variation among them (Figures S5 and S6), even though slight differences were detected based on the group and the model considered (Figure 3, Figures S7 and S8). All the groups showed consistent trends toward more negative values of ΔΨ_tlp_ particularly in the lowlands, although herbaceous angiosperms showed an increasing trend of ΔΨ_tlp_ in the southern part of the study area which encompassed the lagoon (Figure 3c). Gymnosperms displayed a negative trend in the lowland areas, while in the Alps and Prealps the pattern was more variable (Figure 3d), likely due to the larger environmental heterogeneity in these grid cells.

**FIGURE 4:**
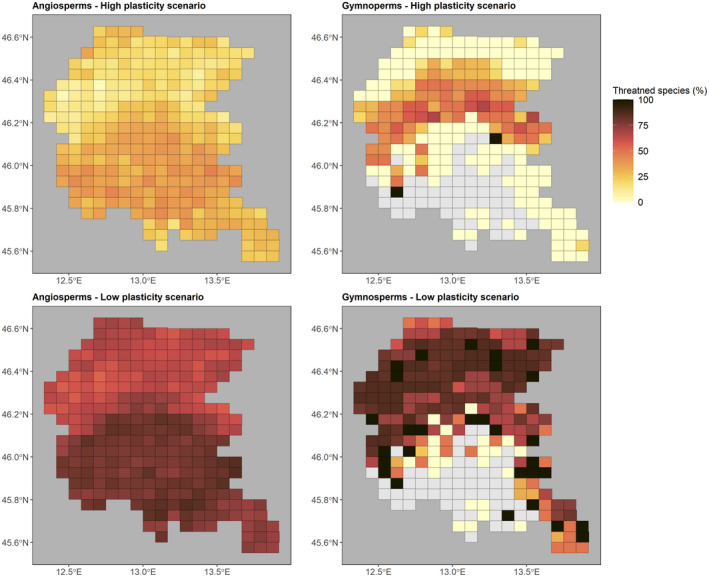
Relative number of species (%) potentially threatened by climate change in the simulated scenarios (high vs. low plasticity) where species were able to adjust their Ψ_tlp_ by 0.44 and 0.22 MPa, respectively. Upper panels represent the high plasticity scenarios for angiosperms (left panels) and gymnosperms (right panels), whereas lower panels represent the lower plasticity scenario. This simulation was performed considering only the high‐emission scenario (RCP 8.5).

The simulations suggested that in the low plasticity scenario, a mean value of 72 ± 8% (mean ± SD) of angiosperm species (49 ± 7% in the high plasticity scenario) within the study area resulted in potential threat by climate change at a local scale. For gymnosperms, 69 ± 28% (19 ± 23% in the high plasticity scenario) of the species in the data set (Figure 4) were at risk. Also detected was a clear spatial pattern of variation, with decreasing Ψ_tlp_ in the lowlands, but increasing Ψ_tlp_ in the mountain areas, with a slight increase in the relative number of threatened species in the low plasticity scenario (Figure S9). In contrast, gymnosperms were mainly threatened by a potential increase in Ψ_tlp_ values in both simulations, particularly where the one simulating a lower plasticity displayed a slight trend consistent with the orography of the study area (Figure S10).

## 4 DISCUSSION

Anticipating the impacts of climate change is one of the greatest challenges of this century. Predicting community‐level responses of terrestrial vegetation by modelling plant water relations and hydraulic traits can inform how species (and ecosystems) will respond to different climatic scenarios. The data show that Ψ_tlp_ can be used for predicting vegetation drought tolerance in mid‐latitude Temperate and Mediterranean biomes. The model also shows that temperature increase and water shortage are expected to induce a shift in the Ψ_tlp_ by approximately 30% compared with current values, and this may ultimately cause the local extinction and displacement of less adaptable/plastic species. Based on the simulations, up to 75% of the species analyzed are at risk to exceed their limits of physiological plasticity in the worst case scenario, exhibiting a lower plasticity in terms of Ψ_tlp_. Detailed Ψ_tlp_ spatialization allowed the upscaling of plant drought resistance to a community scale, indicating the main climatic determinants influencing the spatial patterns of Ψ_tlp_, thus enabling broader generalizations on the potential response of vascular plants to climate change.

### 4.1 Projected shifts of Ψ_tlp_ in relation to climate change

Ψ_tlp_ has recently emerged as a good predictor of plant distribution in response to climatic conditions (Fuchs et al., 2021; Rosas et al., 2019), as it is mechanistically related to drought resistance (Bartlett, Scoffoni, & Sack, 2012; Maréchaux et al., 2018). Specifically, species living in drier environments generally have lower Ψ_tlp_ than species living in areas where water is not a limiting factor (Binks et al., 2016; Fuchs et al., 2021). Ψ_tlp_ is also effective in detecting plant performance under drought conditions or in response to climate change (Kunert et al., 2021; McGregor et al., 2021), whereby species capable of major osmotic adjustments can better withstand water shortage (Binks et al., 2016; Fuchs et al., 2021). In this study, Ψ_tlp_ spatial variation was mainly related to differences in temperature‐related variables, precipitation, and duration of dry spells (Figure 2), which could all be considered as proxies of environmental water availability. Specifically, lower Ψ_tlp_ was found in grid cells with higher temperatures and higher CFD, as well as in those with lower precipitation and higher CDD, confirming previous conclusions on Ψ_tlp_‐environment relationships (Bartlett, Scoffoni, & Sack, 2012; Bourne et al., 2017; Flo et al., 2021; Rosas et al., 2019). The major physiological determinant of Ψ_tlp_ is the cell osmotic potential, which reflects intracellular solute concentration. As a result, species able to decrease their π_0_ under drought can thus lower their Ψ_tlp_ (Bartlett, Scoffoni, & Sack, 2012) and maintain stomatal aperture, photosynthetic gas exchange and growth under water shortage (Blum, 2017; Merchant et al., 2007). In this light, the results confirm that osmoregulation is a key mechanism to cope with both high and low temperature extremes. For example, frost stress can induce water limitation, with possible effects on similar physiological processes affected by drought, such as membrane permeability and stability, water content, and risk of plasmolysis (Charrier, 2021). In this light, the accumulation of solutes (i.e., osmoregulation) is essential to (1) decrease the freezing point in both living cells and apoplast (Charrier et al., 2021; Lintunen et al., 2018) at low temperature extremes and (2) maintain cell hydration by minimizing water loss at high temperature extremes (Chen & Jiang, 2010). Moreover, water stress is exacerbated by the combination of limited water availability and high temperatures in dry and hot areas (such as the coastal and Karst orographic units in the study region, Figure S1), as high temperature increases the evaporative demand of the atmosphere (i.e., higher vapor pressure deficit, VPD), thus increasing water loss through transpiration (Bartlett & Sinclair, 2021). In these areas, reduced water supply and high VPD could cause a drop in xylem pressure below critical thresholds causing embolism formation in xylem conduits, ultimately leading to extreme failure of the water transport system (McDowell et al., 2022). To cope with these conditions, plants can shift physiologically critical thresholds of water status, by decreasing Ψ_tlp_ and/or Ψ_50_ (i.e., water potential inducing 50% loss of hydraulic conductivity) (McDowell et al., 2022). Alternatively, plants can avoid the reduction of xylem pressure by increasing water transport capacity, or by accessing more stable water sources in the soil (e.g., by developing deeper roots), or by decoupling from the atmosphere (e.g., stomata control or leaf area loss) (Kunert et al., 2021; Oliveira et al., 2019; Westoby et al., 2002). In this sense, describing the relationship between plant traits and climate is complicated by the fact that plants can adopt different strategies for coping with seasonal water shortage. Nevertheless, this is fundamental to highlight plant responses to future changes in climate, and the results confirm the importance of Ψ_tlp_ as a functional trait for species and ecosystems description, allowing assessment of their comparative drought tolerance and their potential sensitivity to climate change (Bartlett, Scoffoni, & Sack, 2012).

Future climatic projections portend an increase in frequency and duration of drought events (IPCC, 2021), likely leading to anomalous fluctuations of environmental water availability and possibly threatening plants' survival. In this light, the model predicted future spatial patterns of Ψ_tlp_ in the study area, based on the relationship between current climate and actual distribution of Ψ_tlp_ values. Notably, the direction of Ψ_tlp_ shifts was heterogeneous in the study area. This is highlighted by the different projections emerging in lowlands compared with the Alpine sector. While plant communities at lower elevations will likely undergo a reduction in Ψ_tlp_, a contrasting signal emerged for the Alpine sector, with an apparent shift toward higher Ψ_tlp_. These results suggest that climate change will likely have two major effects on plant communities' composition. On one hand, the projected increase in frequency/intensity of drought events in lowlands will endanger drought‐sensitive species while favoring more resistant ones. The data presented here (Figure 2c,d) indicate that, particularly among the angiosperms, OGUs characterized by more extreme temperature values also display lower Ψ_tlp_. Climate change may amplify temperature stress and eventually mortality, particularly if warmer conditions are associated with prolonged droughts (Fontes et al., 2018), even though the timing of climate change effects can be species‐specific (Vanoni et al., 2016). In contrast, the projected increase of temperature and the reduction of frost days in the Alpine sector, where water availability is not a limiting factor, will likely favor the spread of species positioned toward the fast portion of the acquisitive‐conservative continuum (Petruzzellis et al., 2021; Rosas et al., 2019), which are characterized by lower drought resistance and carbon cost, but higher photosynthetic and growth rates. This agrees with recent research suggesting that prolonged climatic extremes might promote persistent changes in Alpine plant communities and their associated ecosystem functions (De Boeck et al., 2018). Finally, the interplay of drought and frost events might strongly influence water balance and even carbon metabolism, further increasing species vulnerability to climate change (Charrier et al., 2021).

### 4.2 Ecological consequences of projected shifts of Ψ_tlp_ in an accelerated climate change scenario

The sensitivity of species and communities to climate change is strongly influenced by both the intrinsic features of the species (e.g., traits and phylogeny) and the current vegetation type (Fei et al., 2017; Scott et al., 2014). Plants can adjust Ψ_tlp_ in response to fluctuations of soil water availability by accumulating solutes to decrease leaf osmotic potential, or by developing new leaves with higher solute concentrations (Bartlett et al., 2014). However, Ψ_tlp_ plasticity is overall moderate, with an average possible adjustment of 0.44 MPa (Bartlett et al., 2014). Accordingly, the possible future changes of plant communities' composition in the study area will likely depend on the ability of plant species to adjust their Ψ_tlp_ in response to projected climate changes. Based on the simulations, approximately 75% of the species are at risk to exceed the limits of their physiological plasticity in the worst‐case scenario that assumes lower species' plasticity. This is in line with recent studies showing that some of the most iconic European forest species, such as *Fagus sylvatica* and *Picea abies*, are now historically closest to their drought limits (Leuschner, 2020; van der Maaten‐Theunissen et al., 2013). This fact is further corroborated by the steady increase in drought events and associated die‐back events that occurred in Europe over the last decades (Senf et al., 2020), which pose serious threats to the health of European forests. As a result, drier conditions can disrupt local adaptations, promoting upslope migrations (Anderson & Wadgymar, 2020) and rearrangements of species pools, favoring ecological strategies based on drought tolerance or avoidance (Wilcox et al., 2021). Species with higher genetic diversity and interspecific trait variability might cope better with the new conditions (i.e., acclimation) (Choat et al., 2018), but data for both intra‐ and interspecific plasticity of hydraulic traits are still largely absent from the literature (see Tomasella et al., 2018), therefore impeding a finer modelling approach.

Beyond ecological consequences, these changes might also imply modifications of ecosystem services delivered by plant communities. For example, prolonged droughts may increase the occurrence of species that are characterized by harder leaves and more lignified stems, which are usually less digestible by livestock (Lens et al., 2016; Nardini, 2022). Future efforts should focus on monitoring the effects of ongoing global changes on natural vegetation. In this sense, modelling water relation traits, such as leaf turgor loss point over larger spatial extents, while including more species in these analyses, might strongly improve the capacity to effectively anticipate the effects of climate change.

## AUTHOR CONTRIBUTIONS

Enrico Tordoni, Francesco Petruzzellis, Andrea Nardini, and Giovanni Bacaro conceived the study. Enrico Tordoni, Francesco Petruzzellis, and Azzurra Di Bonaventura collected functional trait data; Francesco Boscutti and Fabrizio Martini collected plant community data. Azzurra Di Bonaventura and Martina Tomasella performed the experiment. Enrico Tordoni, Francesco Petruzzellis, Nicola Pavanetto, and Giovanni Bacaro performed the statistical analysis. Enrico Tordoni led the writing of the manuscript with inputs from all the coauthors. All authors approved the submitted version.

## CONFLICT OF INTEREST

The authors declare no competing interests.

## Supporting information


Figure S1
Figure S2
Figure S3
Figure S4
Figure S5
Figure S6
Figure S7
Figure S8
Figure S9
Figure S10
Table S1
Table S2

## Data Availability

The data used in the current study are available in the Zenodo repository: https://doi.org/10.5281/zenodo.6977164.
