# Supplementary material for: Projections of leaf turgor loss point shifts under future climate change scenarios
Source: Glob Chang Biol. 2022 Sep 5;28(22):6640–52. doi: 10.1111/gcb.16400 (PMC9825879; doi:10.1111/gcb.16400)
Supplement: Supplementary file 1 — Figure S1 Figure S2 Figure S3 Figure S4 Figure S5 Figure S6 Figure S7 Figure S8 Figure S9 Figure S10 Table S1 Table S2 [file GCB-28-6640-s001.docx]

**Supporting Information**

Projections of leaf turgor loss point shifts under future climate-change scenarios

Tordoni et al.


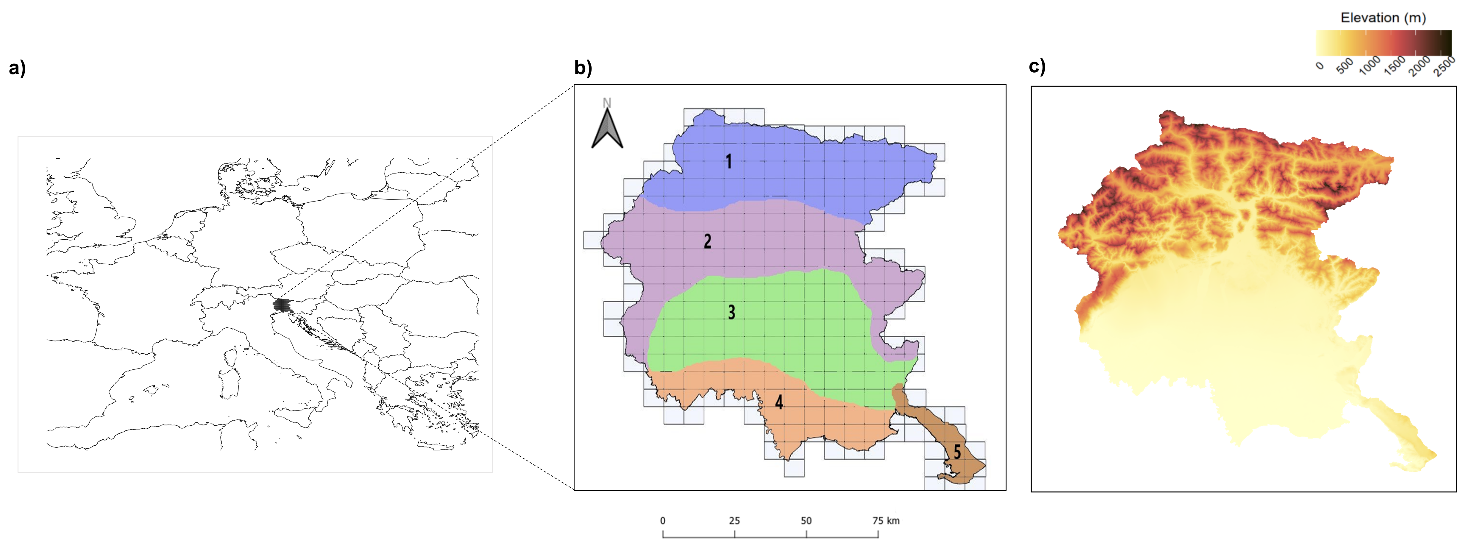


**Figure S1.** a) Location of Friuli Venezia Giulia region (NE Italy) with respect to European continent. B) Map of the five areas reflecting the main orographic units in the region (1=Alps, 2=Pre-Alps, 3=High Plains, 4=Low Plains and coast, 5=classic Karst) on which grid cells were overlaid, and c) map of elevation of the study area.





**Figure S2.** Boxplots reporting the variation of leaf turgor loss point - Ψ_tlp_ for each habitat considered in this study. Different letters denote significant differences based on Tukey’s HSD test (alpha = 0.05).


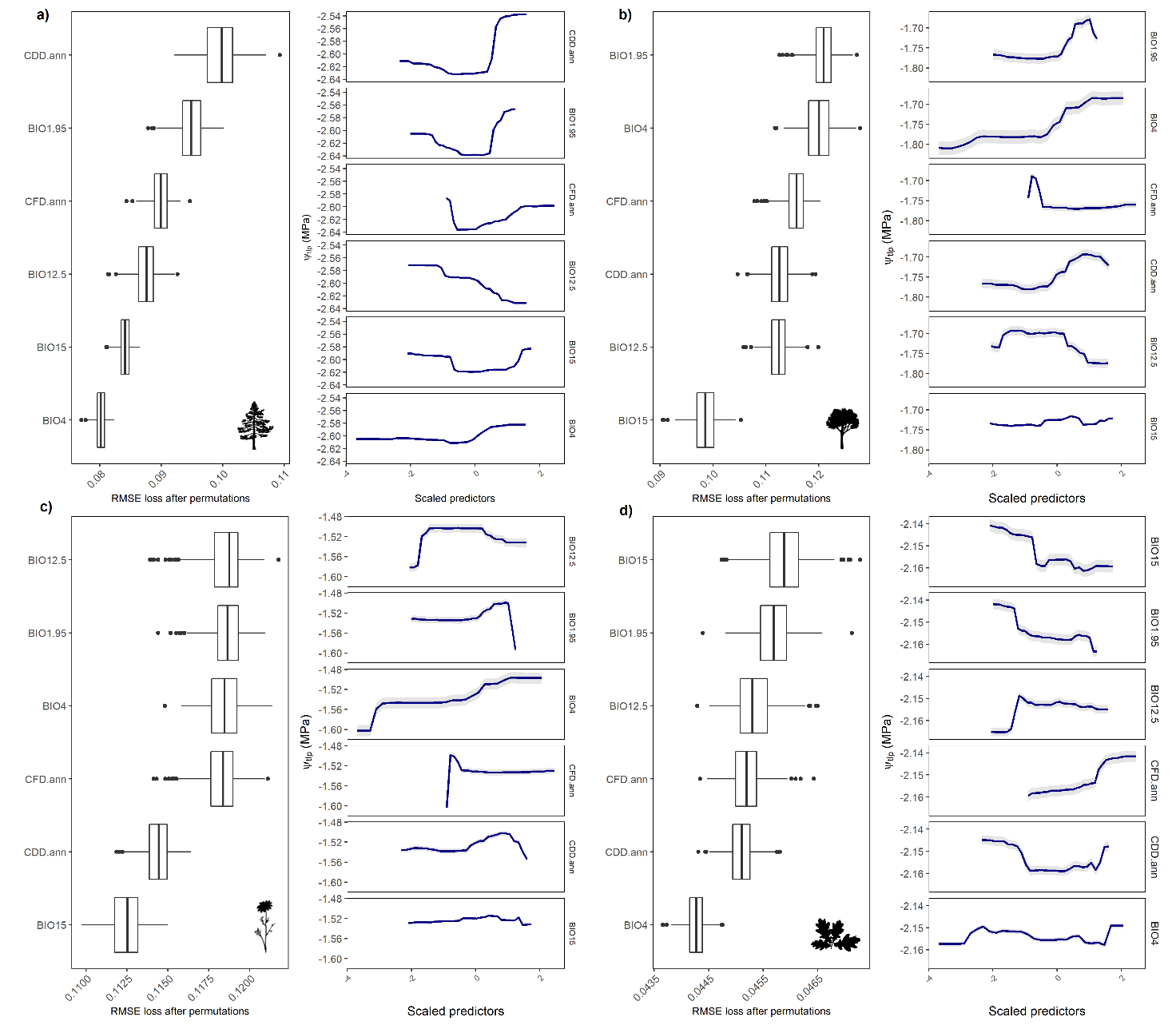


**Figure S3.** Variable importance (left panel) and marginal effects (right panel) related to the model EC-EARTH_CCLM4-8-17 for a) Gymnosperms, b) Angiosperms, c) Herbaceous angiosperms, d) Woody angiosperms. Boxplots showed variable importance ranked by the RMSE loss after permutations while solid lines are marginal effects (mean ± 2 SE). BIO1.95 = 95^th^ percentiles of average temperature, BIO4 = temperature seasonality, CFD.ann = annual consecutive frost days where temperature was ≤ 0 °C, CDD.ann = annual consecutive dry days where precipitation was < 1 mm, BIO12.5 = 5^th^ percentiles of cumulate annual precipitation, BIO15 = precipitation seasonality. Please note that all predictors have been centered and scaled to unit variance. Silhouettes were retrieved from http://phylopic.org.


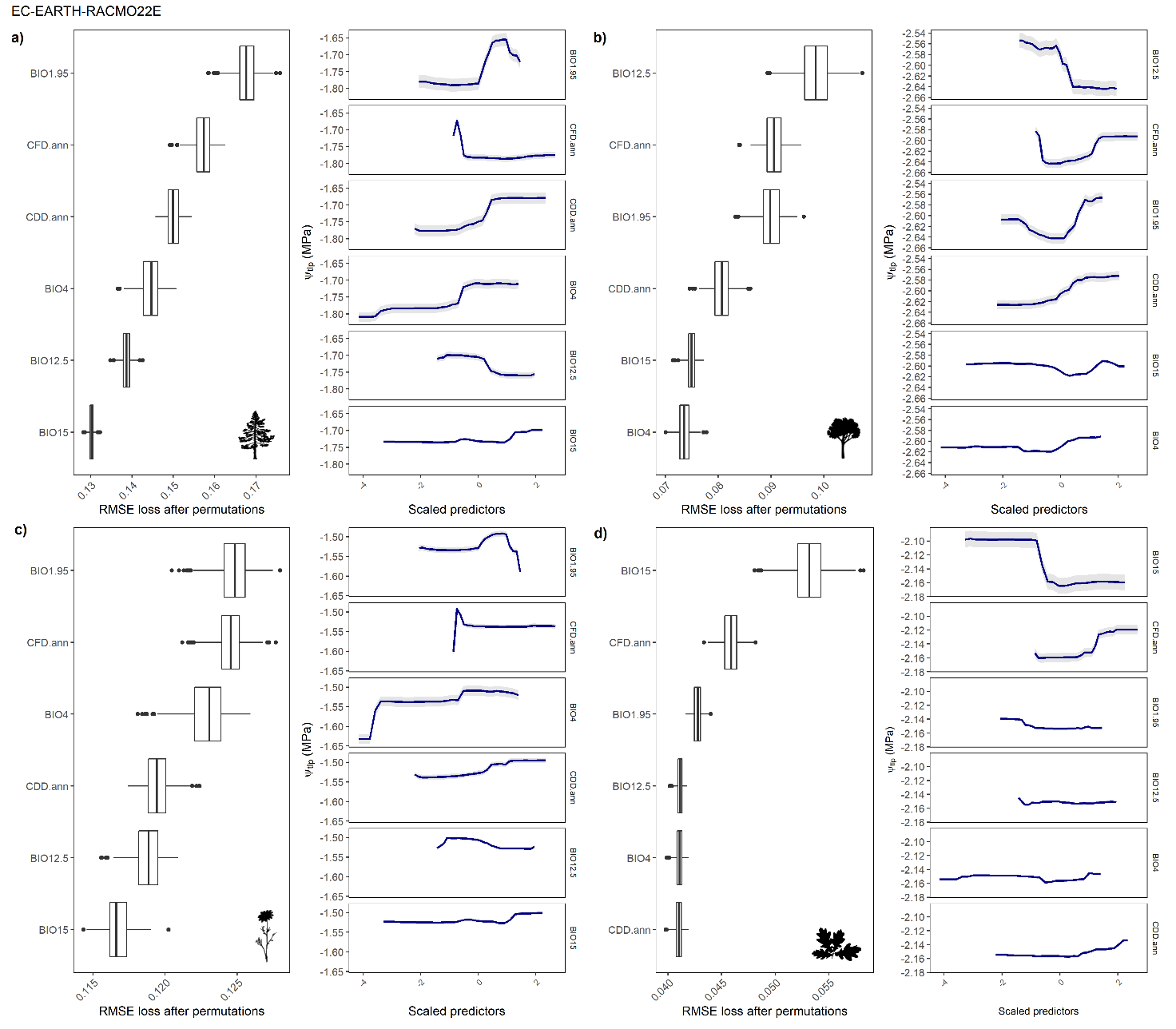


**Figure S4.** Variable importance (left panel) and marginal effects (right panel) related to the model EC-EARTH_RACMO22E for a) Gymnosperms, b) Angiosperms, c) Herbaceous angiosperms, d) Woody angiosperms. Boxplots showed variable importance ranked by the RMSE loss after permutations while solid lines are marginal effects (mean ± 2 SE). BIO1.95 = 95^th^ percentiles of average temperature, BIO4 = temperature seasonality, CFD.ann = annual consecutive frost days where temperature was ≤ 0 °C, CDD.ann = annual consecutive dry days where precipitation was < 1 mm, BIO12.5 = 5^th^ percentiles of cumulate annual precipitation, BIO15 = precipitation seasonality. Please note that all predictors have been centered and scaled to unit variance. Silhouettes were retrieved from <http://phylopic.org>.


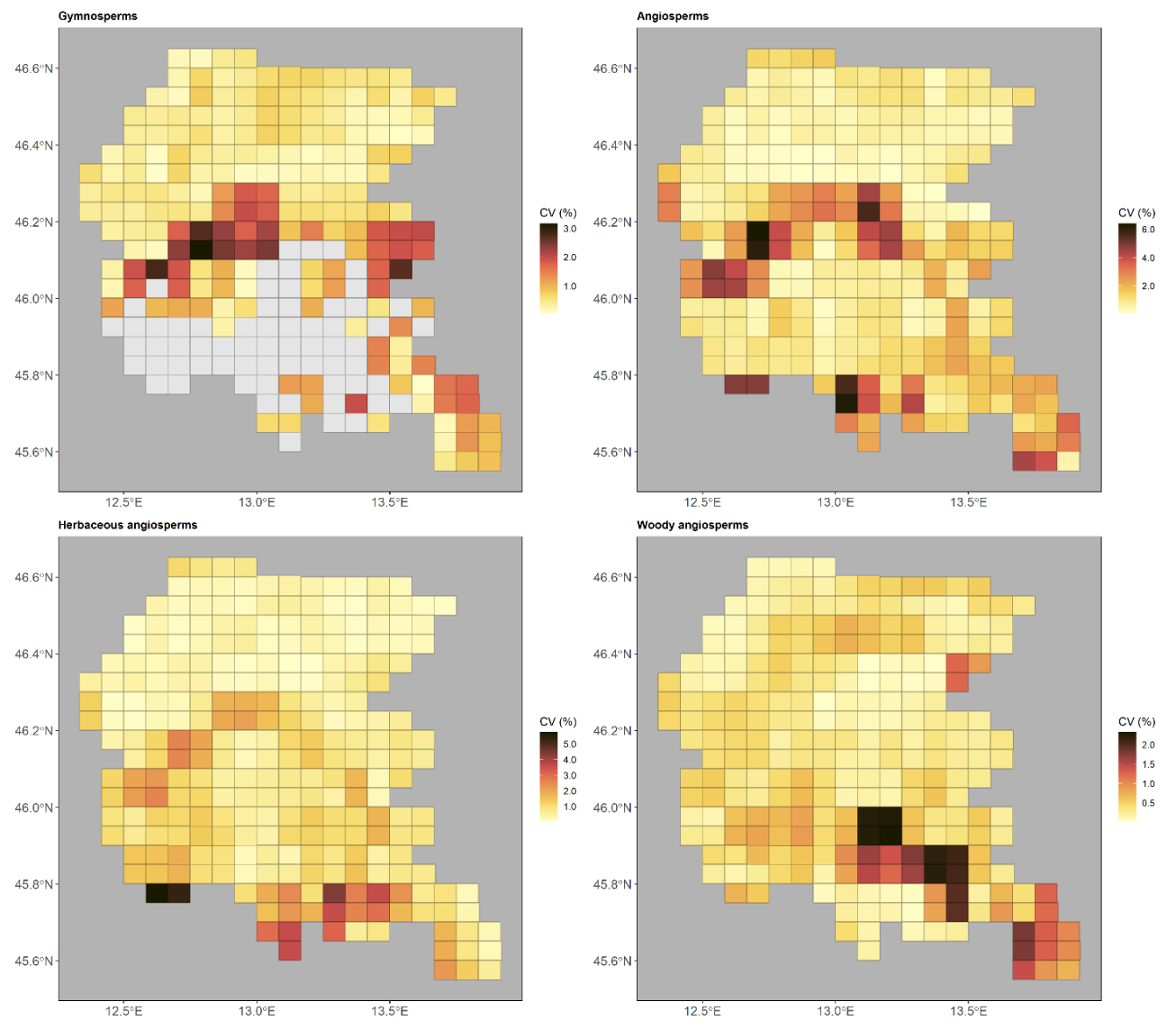


**Figure S5.** Maps reporting the coefficient of variation (CV, %) of the model prediction among the three models in the scenario RCP 2.6. Darker tones indicate higher variation among predictions and vice versa.


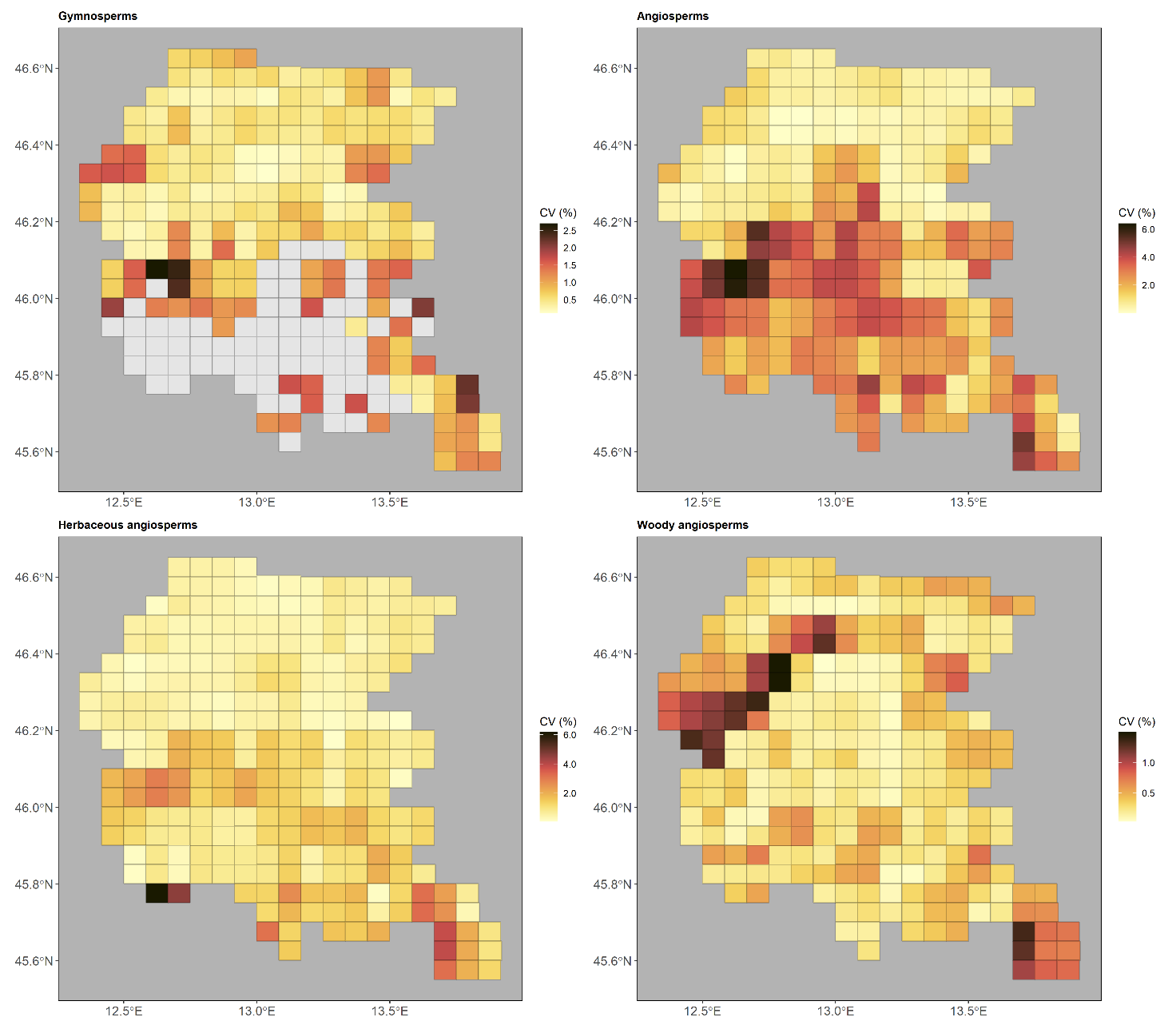


**Figure S6.** Maps reporting the coefficient of variation (CV, %) of the model prediction among the three models in the scenario RCP 8.5. Darker tones indicate higher variation among predictions and vice versa.


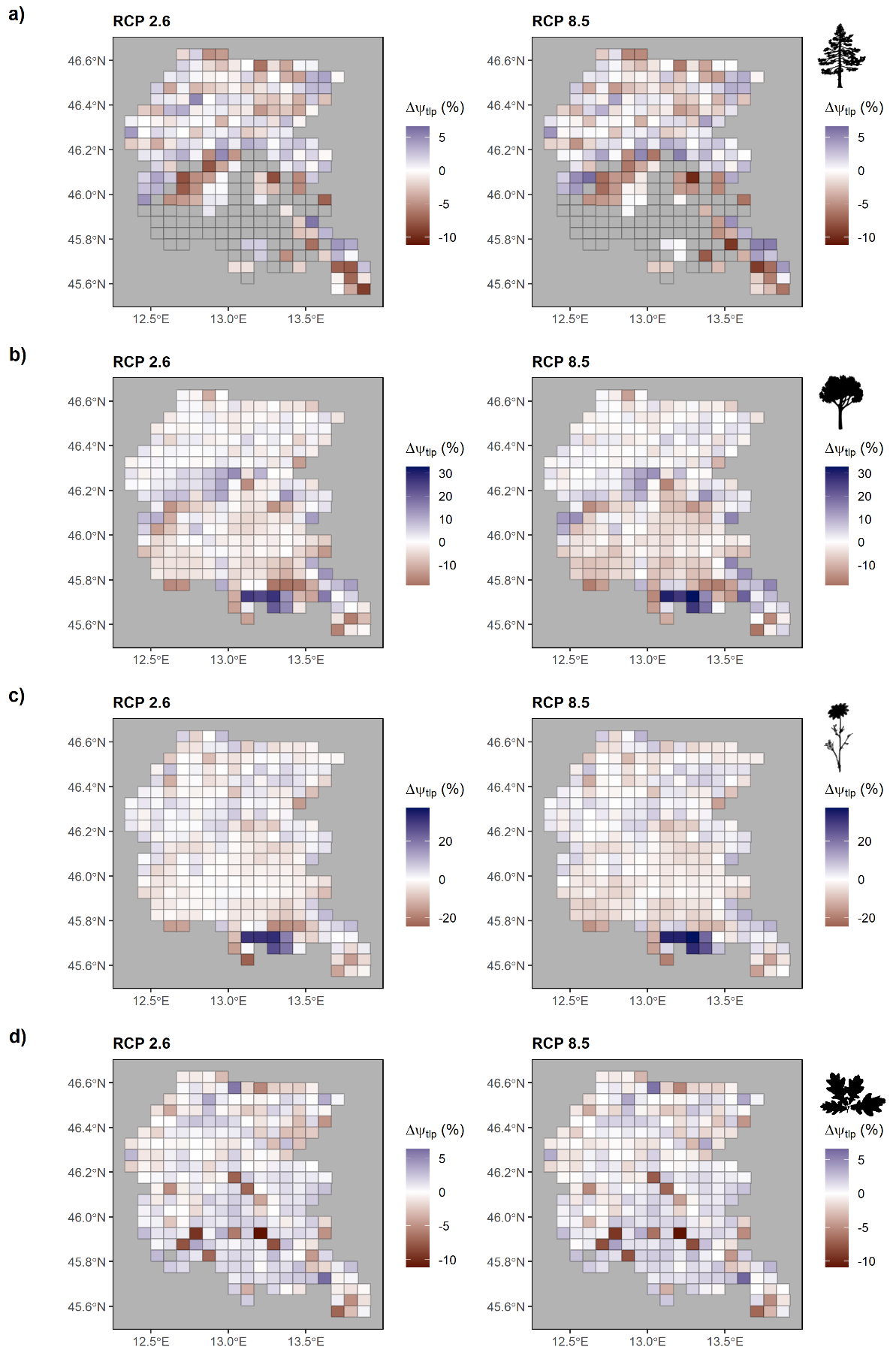


**Figure S7.** Projections of turgor loss point shifts (ΔΨ_tlp_, %) based on two RCP scenarios (RCP 2.6 and RCP 8.5) related to the model EC-EARTH_CCLM4-8-17 for a) gymnosperms, b) angiosperms, c) herbaceous angiosperms, and d) woody angiosperms. Brown tones indicate a shift toward more negative values of Ψ_tlp_ and bluish tones denote a shift toward higher values of Ψ_tlp_. Silhouettes were retrieved from <http://phylopic.org>.

**
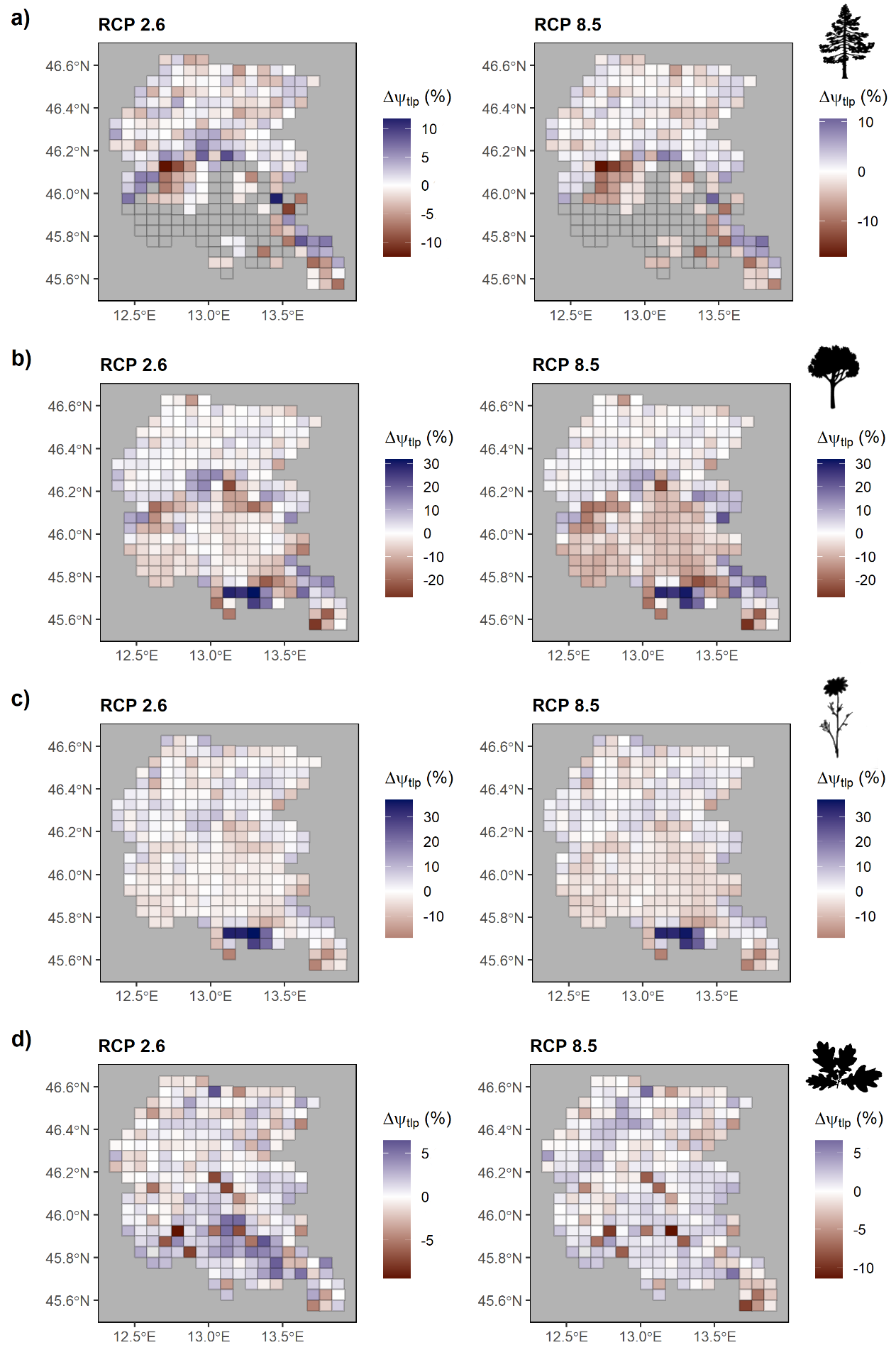
**

**Figure S8.** Projections of turgor loss point shifts (ΔΨ_tlp_, %) based on two RCP scenarios (RCP 2.6 and RCP 8.5) related to the model EC-EARTH_RACMO22E for a) gymnosperms, b) angiosperms, c) herbaceous angiosperms, and d) woody angiosperms. Brown tones indicate a shift toward more negative values of Ψ_tlp_ and bluish tones denote a shift toward higher values of Ψ_tlp_. Silhouettes were retrieved from <http://phylopic.org>.


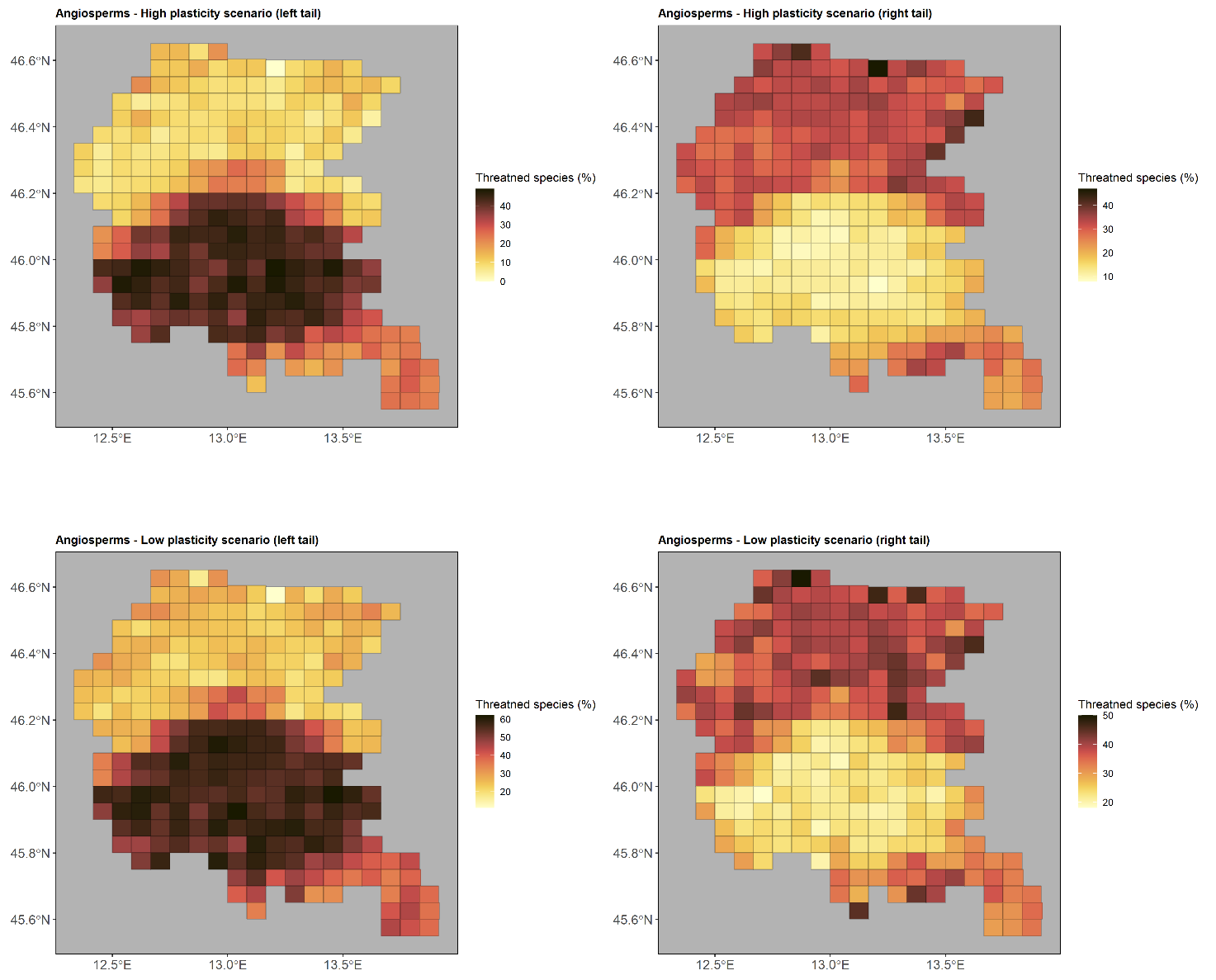


**Figure S9.** Relative number of species (%) potentially threatened by climate change within angiosperms in the simulated scenarios (high vs low plasticity) where species were able to adjust their Ψ_tlp_ by 0.44 MPa and 0.22 MPa, respectively. Upper panels represent the high plasticity scenarios considering only negative shifts (left panel) and positive ones (right panel), while lower panels represented the low plasticity scenario. Please note that this simulation was performed considering only the high-emission scenario (RCP 8.5).


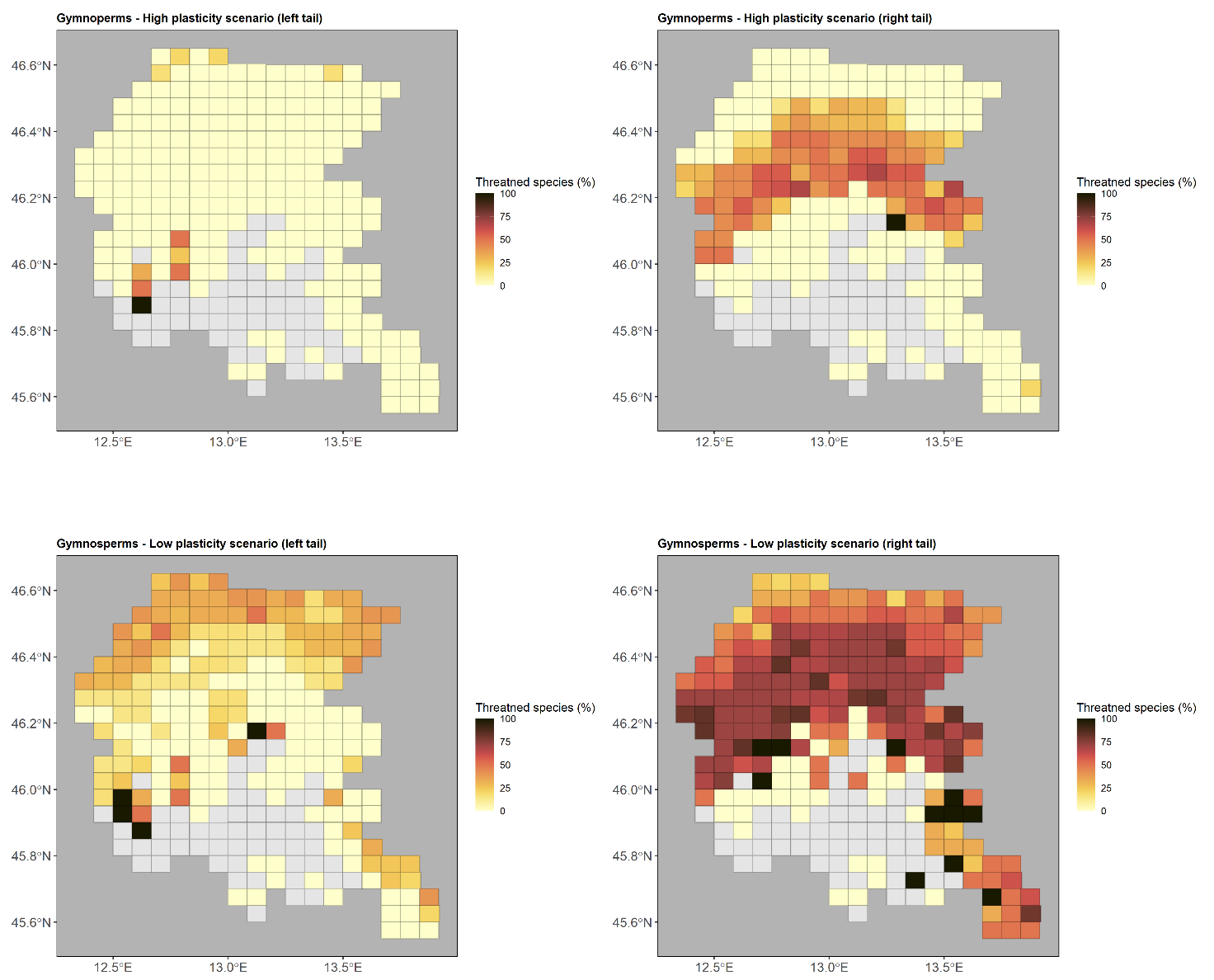


**Figure S10.** Relative number of species (%) potentially threatened by climate change within angiosperms in the simulated scenarios (high vs low plasticity) where species were able to adjust their Ψ_tlp_ by 0.44 MPa and 0.22 MPa, respectively. Upper panels represent the high plasticity scenarios considering only negative shifts (left panel) and positive ones (right panel), while lower panels represented the low plasticity scenario. Please note that this simulation was performed considering only the high-emission scenario (RCP 8.5).

**Tables**

**Table S1.** List of species used in this study. Species names were standardized following the updated checklist of Italian vascular flora (Bartolucci et al. 2018).

| **Species** | **Clade** | **Growth form** |
| --- | --- | --- |
| Abies alba Mill. | Gymnosperm | Woody |
| Acer campestre L. | Angiosperm | Woody |
| Acer monspessulanum L. subsp. monspessulanum | Angiosperm | Woody |
| Acer platanoides L. | Angiosperm | Woody |
| Acer pseudoplatanus L. | Angiosperm | Woody |
| Agrostis stolonifera L. subsp. stolonifera | Angiosperm | Herbaceous |
| Ailanthus altissima (Mill.) Swingle | Angiosperm | Woody |
| Alkekengi officinarum Moench | Angiosperm | Herbaceous |
| Allium sphaerocephalon L. subsp. sphaerocephalon | Angiosperm | Herbaceous |
| Ambrosia artemisiifolia L. | Angiosperm | Herbaceous |
| Ambrosia psilostachya DC. | Angiosperm | Herbaceous |
| Amorpha fruticosa L. | Angiosperm | Woody |
| Anthyllis vulneraria L. | Angiosperm | Herbaceous |
| Aristolochia clematitis L. | Angiosperm | Herbaceous |
| Arrhenatherum elatius (L.) P.Beauv. ex J.Presl & C.Presl subsp. elatius | Angiosperm | Herbaceous |
| Atriplex prostrata Boucher ex DC. | Angiosperm | Herbaceous |
| Betula pendula Roth | Angiosperm | Woody |
| Bidens frondosa L. | Angiosperm | Herbaceous |
| Bidens vulgata Greene | Angiosperm | Herbaceous |
| Bolboschoenus maritimus (L.) Palla | Angiosperm | Herbaceous |
| Brachypodium sylvaticum (Huds.) P.Beauv. | Angiosperm | Herbaceous |
| Bromopsis erecta (Huds.) Fourr. | Angiosperm | Herbaceous |
| Cakile maritima Scop. subsp. maritima | Angiosperm | Herbaceous |
| Calamagrostis arenaria (L.) Roth subsp. arundinacea (Husn.) Banfi, Galasso & Bartolucci | Angiosperm | Herbaceous |
| Calamagrostis epigejos (L.) Roth subsp. epigejos | Angiosperm | Herbaceous |
| Carex alba Scop. | Angiosperm | Herbaceous |
| Carex elata All. subsp. elata | Angiosperm | Herbaceous |
| Carex extensa Gooden. | Angiosperm | Herbaceous |
| Carex humilis Leyss. | Angiosperm | Herbaceous |
| Carex vesicaria L. | Angiosperm | Herbaceous |
| Carpinus betulus L. | Angiosperm | Woody |
| Carpinus orientalis Mill. subsp. orientalis | Angiosperm | Woody |
| Castanea sativa Mill. | Angiosperm | Woody |
| Celtis australis L. | Angiosperm | Woody |
| Cenchrus longispinus (Hack.) Fernald | Angiosperm | Herbaceous |
| Centaurea rupestris L. | Angiosperm | Herbaceous |
| Chrysopogon gryllus (L.) Trin. | Angiosperm | Herbaceous |
| Cichorium intybus L. | Angiosperm | Herbaceous |
| Clematis vitalba L. | Angiosperm | Herbaceous |
| Cornus mas L. | Angiosperm | Woody |
| Cornus sanguinea L. | Angiosperm | Woody |
| Corylus avellana L. | Angiosperm | Woody |
| Cotinus coggygria Scop. | Angiosperm | Woody |
| Crataegus monogyna Jacq. | Angiosperm | Woody |
| Cynodon dactylon (L.) Pers. | Angiosperm | Herbaceous |
| Cyperus capitatus Vand. | Angiosperm | Herbaceous |
| Cytisus pseudoprocumbens Markgr. | Angiosperm | Herbaceous |
| Dictamnus albus L. | Angiosperm | Herbaceous |
| Elymus acutus (DC.) M.A.Thiébaud | Angiosperm | Herbaceous |
| Elymus farctus (Viv.) Runemark ex Melderis | Angiosperm | Herbaceous |
| Elymus repens (L.) Gould subsp. repens | Angiosperm | Herbaceous |
| Erica carnea L. subsp. carnea | Angiosperm | Woody |
| Erigeron annuus (L.) Desf. | Angiosperm | Herbaceous |
| Erigeron canadensis L. | Angiosperm | Herbaceous |
| Eryngium amethystinum L. | Angiosperm | Herbaceous |
| Eryngium maritimum L. | Angiosperm | Herbaceous |
| Euphorbia cyparissias L. | Angiosperm | Herbaceous |
| Fagus sylvatica L. subsp. sylvatica | Angiosperm | Woody |
| Fraxinus excelsior L. subsp. excelsior | Angiosperm | Woody |
| Fraxinus ornus L. subsp. ornus | Angiosperm | Woody |
| Galium palustre L. subsp. palustre | Angiosperm | Herbaceous |
| Galium verum L. | Angiosperm | Herbaceous |
| Geum urbanum L. | Angiosperm | Herbaceous |
| Gratiola officinalis L. | Angiosperm | Herbaceous |
| Hainardia cylindrica (Willd.) Greuter | Angiosperm | Herbaceous |
| Halimione portulacoides (L.) Aellen | Angiosperm | Herbaceous |
| Hedera helix L. subsp. helix | Angiosperm | Herbaceous |
| Helleborus niger L. | Angiosperm | Herbaceous |
| Hepatica nobilis Mill. | Angiosperm | Herbaceous |
| Hippocrepis comosa L. subsp. comosa | Angiosperm | Herbaceous |
| Juncus maritimus Lam. | Angiosperm | Herbaceous |
| Juniperus communis L. | Gymnosperm | Woody |
| Jurinea mollis (L.) Rchb. subsp. mollis | Angiosperm | Herbaceous |
| Koeleria pyramidata (Lam.) P.Beauv. | Angiosperm | Herbaceous |
| Larix decidua Mill. | Gymnosperm | Woody |
| Leersia oryzoides (L.) Sw. | Angiosperm | Herbaceous |
| Limbarda crithmoides (L.) Dumort. subsp. longifolia (Arcang.) Greuter | Angiosperm | Herbaceous |
| Iris pseudacorus (L.) Fuss | Angiosperm | Herbaceous |
| Limonium narbonense Mill. | Angiosperm | Herbaceous |
| Lotus corniculatus L. subsp. corniculatus | Angiosperm | Herbaceous |
| Lotus germanicus (Gremli) Peruzzi | Angiosperm | Herbaceous |
| Luzula luzuloides (Lam.) Dandy & Wilmott | Angiosperm | Herbaceous |
| Lysimachia vulgaris L. | Angiosperm | Herbaceous |
| Lythrum salicaria L. | Angiosperm | Herbaceous |
| Medicago falcata L. subsp. falcata | Angiosperm | Herbaceous |
| Melica ciliata L. subsp. ciliata | Angiosperm | Herbaceous |
| Mentha arvensis L. | Angiosperm | Herbaceous |
| Nardus stricta L. | Angiosperm | Herbaceous |
| Oenothera stucchii Soldano | Angiosperm | Herbaceous |
| Ornithogalum kochii Parl. | Angiosperm | Herbaceous |
| Ostrya carpinifolia Scop. | Angiosperm | Woody |
| Oxalis dillenii Jacq. | Angiosperm | Herbaceous |
| Persicaria dubia (Stein.) Fourr. | Angiosperm | Herbaceous |
| Persicaria hydropiper (L.) Delarbre | Angiosperm | Herbaceous |
| Phragmites australis (Cav.) Trin. ex Steud. | Angiosperm | Herbaceous |
| Phyteuma spicatum L. subsp. spicatum | Angiosperm | Herbaceous |
| Picea abies (L.) H. Karst. | Gymnosperm | Woody |
| Pinus mugo Turra subsp. mugo | Gymnosperm | Woody |
| Pinus nigra J.F.Arnold subsp. nigra | Gymnosperm | Woody |
| Pinus sylvestris L. | Gymnosperm | Woody |
| Pistacia terebinthus L. subsp. terebinthus | Angiosperm | Woody |
| Plantago major L. | Angiosperm | Herbaceous |
| Plantago subulata L. | Angiosperm | Herbaceous |
| Poa alpina L. subsp. alpina | Angiosperm | Herbaceous |
| Polygala comosa Schkuhr | Angiosperm | Herbaceous |
| Populus nigra L. subsp. nigra | Angiosperm | Woody |
| Populus tremula L. | Angiosperm | Woody |
| Potentilla reptans L. | Angiosperm | Herbaceous |
| Poterium sanguisorba L. | Angiosperm | Herbaceous |
| Primula vulgaris Huds. subsp. vulgaris | Angiosperm | Herbaceous |
| Prunus mahaleb L. subsp. mahaleb | Angiosperm | Woody |
| Quercus ilex L. subsp. ilex | Angiosperm | Woody |
| Quercus petraea (Matt.) Liebl. subsp. petraea | Angiosperm | Woody |
| Quercus pubescens Willd. subsp. pubescens | Angiosperm | Woody |
| Quercus robur L. | Angiosperm | Woody |
| Ranunculus lanuginosus L. | Angiosperm | Herbaceous |
| Ranunculus repens L. | Angiosperm | Herbaceous |
| Rhamnus cathartica L. | Angiosperm | Woody |
| Rhododendron ferrugineum L. | Angiosperm | Woody |
| Rhododendron hirsutum L. | Angiosperm | Woody |
| Robinia pseudoacacia L. | Angiosperm | Woody |
| Rorippa sylvestris (L.) Besser subsp. sylvestris | Angiosperm | Herbaceous |
| Rubus caesius L. | Angiosperm | Woody |
| Rubus ulmifolius Schott | Angiosperm | Woody |
| Ruscus aculeatus L. | Angiosperm | Herbaceous |
| Salicornia fruticosa (L.) L. | Angiosperm | Herbaceous |
| Salix eleagnos Scop. | Angiosperm | Woody |
| Salsola tragus L. | Angiosperm | Herbaceous |
| Salvia pratensis L. | Angiosperm | Herbaceous |
| Sambucus nigra L. | Angiosperm | Woody |
| Satureja montana L. subsp. montana | Angiosperm | Herbaceous |
| Scabiosa triandra L. | Angiosperm | Herbaceous |
| Schoenus nigricans L. | Angiosperm | Herbaceous |
| Scorzonera villosa Scop. subsp. villosa | Angiosperm | Herbaceous |
| Senecio inaequidens DC. | Angiosperm | Herbaceous |
| Sesleria autumnalis (Scop.) F.W.Schultz | Angiosperm | Herbaceous |
| Setaria italica (L.) P.Beauv. subsp. viridis (L.) Thell. | Angiosperm | Herbaceous |
| Silene vulgaris (Moench) Garcke subsp. vulgaris | Angiosperm | Herbaceous |
| Soda inermis Fourr. | Angiosperm | Herbaceous |
| Sonchus oleraceus L. | Angiosperm | Herbaceous |
| Sorbus aria (L.) Crantz | Angiosperm | Woody |
| Sorbus aucuparia L. subsp. aucuparia | Angiosperm | Woody |
| Sorghum halepense (L.) Pers. | Angiosperm | Herbaceous |
| Sporobolus pumilus (Roth) P.M.Peterson & Saarela | Angiosperm | Herbaceous |
| Stipa pennata L. subsp. pennata | Angiosperm | Herbaceous |
| Suaeda maritima (L.) Dumort. | Angiosperm | Herbaceous |
| Tanacetum corymbosum (L.) Sch.Bip. | Angiosperm | Herbaceous |
| Teucrium chamaedrys L. subsp. chamaedrys | Angiosperm | Herbaceous |
| Teucrium montanum L. | Angiosperm | Herbaceous |
| Teucrium scordium L. subsp. scordium | Angiosperm | Herbaceous |
| Thalictrum flavum L. | Angiosperm | Herbaceous |
| Thalictrum minus L. subsp. minus | Angiosperm | Herbaceous |
| Thymus pulegioides L. | Angiosperm | Herbaceous |
| Tilia platyphyllos Scop. | Angiosperm | Woody |
| Trachomitum venetum (L.) Woodson subsp. venetum | Angiosperm | Woody |
| Trifolium pratense L. | Angiosperm | Herbaceous |
| Trinia glauca (L.) Dumort. | Angiosperm | Herbaceous |
| Tripidium ravennae (L.) H.Scholz subsp. ravennae | Angiosperm | Herbaceous |
| Ulmus glabra Huds. | Angiosperm | Woody |
| Ulmus minor Mill. subsp. minor | Angiosperm | Woody |
| Urtica dioica L. subsp. dioica | Angiosperm | Herbaceous |
| Vaccinium myrtillus L. | Angiosperm | Woody |
| Verbascum densiflorum Bertol. | Angiosperm | Herbaceous |
| Vincetoxicum hirundinaria Medik. subsp. hirundinaria | Angiosperm | Herbaceous |
| Xanthium orientale L. | Angiosperm | Herbaceous |
| Yucca gloriosa L. | Angiosperm | Woody |

**Table S2.** Model performance based on spatial cross validation in the four groups considered in this study (gymnosperms, angiosperms, herbaceous and woody angiosperms). RMSE= Root Mean Square Error.

| **Group** | **HadGEM2-ES_RACMO22E** | | **EC-EARTH_RACMO22E** | | **EC-EARTH_CCLM4-8-17** | |
| --- | --- | --- | --- | --- | --- | --- |
|  | RMSE | R^2^ | RMSE | R^2^ | RMSE | R^2^ |
| Gymnosperms | 0.11 ± 0.04 | 0.58 ±0.05 | 0.12 ±0.04 | 0.58 ±0.05 | 0.11 ±0.04 | 0.64 ±0.05 |
| Angiosperms | 0.16 ± 0.06 | 0.70 ±0.04 | 0.18 ±0.06 | 0.67 ±0.04 | 0.14 ±0.06 | 0.70 ±0.02 |
| Herbaceous angiosperms | 0.10 ± 0.07 | 0.44 ±0.06 | 0.12 ±0.08 | 0.40 ±0.04 | 0.08 ±0.07 | 0.43 ±0.04 |
| Woody angiosperms | 0.05 ± 0.01 | 0.25 ±0.08 | 0.05 ±0.01 | 0.25 ±0.07 | 0.05 ±0.01 | 0.25 ±0.08 |
